# A Bayesian Approach to the Estimation of Parameters and Their Interdependencies in Environmental Modeling †

**DOI:** 10.3390/e24020231

**Published:** 2022-02-03

**Authors:** Christopher G. Albert, Ulrich Callies, Udo von Toussaint

**Affiliations:** 1 Max-Planck-Institut für Plasmaphysik, 85748 Garching, Germany; udt@ipp.mpg.de; 2 Institute of Theoretical and Computational Physics, Technische Universität Graz, 8010 Graz, Austria; 3 Helmholtz-Zentrum Hereon, 21502 Geesthacht, Germany; ulrich.callies@hereon.de

**Keywords:** model calibration, overparameterization, posterior parameter dependence, Markov chain Monte Carlo, delayed acceptance, Bayesian network

## Abstract

We present a case study for Bayesian analysis and proper representation of distributions and dependence among parameters when calibrating process-oriented environmental models. A simple water quality model for the Elbe River (Germany) is referred to as an example, but the approach is applicable to a wide range of environmental models with time-series output. Model parameters are estimated by Bayesian inference via Markov Chain Monte Carlo (MCMC) sampling. While the best-fit solution matches usual least-squares model calibration (with a penalty term for excessive parameter values), the Bayesian approach has the advantage of yielding a joint probability distribution for parameters. This posterior distribution encompasses all possible parameter combinations that produce a simulation output that fits observed data within measurement and modeling uncertainty. Bayesian inference further permits the introduction of prior knowledge, e.g., positivity of certain parameters. The estimated distribution shows to which extent model parameters are controlled by observations through the process of inference, highlighting issues that cannot be settled unless more information becomes available. An interactive interface enables tracking for how ranges of parameter values that are consistent with observations change during the process of a step-by-step assignment of fixed parameter values. Based on an initial analysis of the posterior via an undirected Gaussian graphical model, a directed Bayesian network (BN) is constructed. The BN transparently conveys information on the interdependence of parameters after calibration. Finally, a strategy to reduce the number of expensive model runs in MCMC sampling for the presented purpose is introduced based on a newly developed variant of delayed acceptance sampling with a Gaussian process surrogate and linear dimensionality reduction to support function-valued outputs.

## 1. Introduction

Mathematical ecosystem models differ with regard to mathematical complexity and the number of free parameters involved. Even the most complex models vastly simplify reality, including arbitrary choices with respect to their structure. It remains as an ever-present challenge to suitably balance model complexity with the amount of data available for model calibration. Having agreed on a certain structure, overparameterization, i.e., parameters not being controlled by observational evidence, is a ubiquitous problem. Different parameter combinations may produce very similar results, e.g., [1,2,3,4,5]. Simultaneously strengthening one process and weakening another may have no substantial overall effects. As a result, some parameters can be set to unrealistic values without contradiction with the data under study.

From a purely mathematical point of view, the problem of overparameterization should be alleviated by striving for a reduced number of model parameters, discarding simulation of details. Alternatively, variables might be aggregated into a set of few compound variables. However, lumped parameters often lack a clear process-oriented interpretation. A simplified pure input–output model might be successful in predicting variables of interest but can usually not explain why certain things are going to happen. In process-oriented simulations, each model parameter has a specific meaning. Knowing about its interpretation will be important for any informed management action based on integrated assessments [6]. The method proposed in this study therefore keeps the full set of model parameters. However, instead of providing for each of these parameters its most probable value (possibly together with an error bar), we provide a description of the joint probability density of the full set of parameters.

We tackle model calibration by methods of Bayesian probability theory (see, e.g., [7,8] for an introduction). This theory provides a consistent framework of assigning probabilities to quantify a degree of belief. In contrast to frequentist analysis, where probability is usually defined in terms of countable statistics, Bayesian analysis enables the assignment of probabilities to sets of model parameters before (prior) and after (posterior) the observation of calibration data, using the likelihood that the model generates these data. Investigating the structure of the posterior distribution sheds light on the quality of the estimation of model parameters and their interdependencies by observational data.

We propose using the Bayesian network (BN) technology [9,10] to describe interdependencies within parameter sets that produce simulations in agreement with observed data. In contrast to their established use for statistical modeling, we apply BNs to the analysis of a posterior after calibrating a process model. The underlying ensemble of successful simulations is produced performing Markov Chain Monte Carlo (MCMC) simulations. A BN represents a joint distribution of multivariate data by its factorization in terms of conditional probabilities. Various software packages are available for that purpose. Evidence provided for any subset of parameters can be spread across the whole network, potentially changing the marginal distributions of all other parameters.

The problem with constructing a saturated BN (retaining all possible interactions) is the dimension and overall size of conditional probability tables needed. However, as long as all parameters are allowed to interact, conditional marginal distributions can also be obtained by sub-sampling from the set of successful parameter sets generated by MCMC. In a first step, we will follow this direct approach. A benefit from using a BN based on conditional probability tables fitted to the data arises when the goal is to focus on just the most important interaction patterns. A BN displays such interaction structure in terms of a directed acyclic graph (DAG). Often, a DAG is seen as a means to represent cause–effect relationships [11,12]. Although this concept is not applicable for the example under study, we explore graph simplification to visualize key parameter dependencies.

An important limitation of the presented approach is the required computing time for simulation runs during MCMC sampling. Delayed acceptance can accelerate the procedure up to a factor of one over the acceptance rate [13,14]. In order to do so, it requires a surrogate of the posterior that contains the cost function inside the likelihood in case of model calibration. The simplest way to implement delayed acceptance relies on a surrogate with scalar output built for this cost function or for the likelihood. Here, we take an intermediate step and construct a surrogate for the functional output of a blackbox model to be calibrated against reference data. Typical examples are numerical simulations that output time series or spatial data and depend on tunable input parameters. We demonstrate the application of this approach on two examples using usual and hierarchical Bayesian model calibration. In the latter case, a surrogate beyond the $L_{2}$ cost function is required if the likelihood depends on additional auxiliary parameters. As an example, we allow variations of the (fractional) order of the norm, thereby marginalizing over different noise models, including Gaussian and Laplacian noise.

This paper is organized in the following way. In Section 2, this work is put into context with existing literature. In Section 3, we describe the problem addressed as a case study, a simple numerical model that simulates chlorophyll *a* time series observed at a station on the Elbe River. Section 4 gives a short introduction into Bayesian probability theory and the way Markov Chain Monte Carlo is implemented in the context of model calibration. A brief introduction is given to graphical modeling, encompassing both Gaussian graphical models and Bayesian networks. A special tool for analyzing conditional posterior marginal distributions of MCMC parameter samples is presented. In Section 5, we first illustrate model output uncertainties that arise from posterior model parameter uncertainties. Then, dependencies between calibrated parameters are explored, looking at conditionalized marginal distributions. Finally, a Bayesian network with simplified parameter dependencies is devised based on the results from fitting a Gaussian graphical model to the MCMC parameter samples. Section 6 provides a comprehensive discussion followed by some conclusions.

## 2. Relation to Existing Work

In the light of inevitable model uncertainties, Fedra [15] proposed a replacement of predictions that pretend an unrealistic precision by multiple predictions covering predictive uncertainty. He suggested models be used for more qualitative discrimination between different options rather than for detailed predictions. Identifiability of model parameters needs the observed part of model output to be sensitive to these parameters [16]. However, lacking identifiability must be distinguished from model output insensitivity [17]. Local sensitivity analyses based on local derivatives of some model output are suitable when a model is essentially linear. By contrast, global sensitivity analysis (GSA) takes a sampling approach to apportion model output uncertainties to uncertainties of single input parameters or combinations thereof [18]. Model output variance may be decomposed in terms of orthogonal partial variances with an increasing number of input parameters contributing to them (Sobol’ indices; [19]). In practice, the large number of Monte Carlo simulations needed often makes calculation of higher order partial variances infeasible. Sudret [20] proposes the use of surrogate models based on polynomial chaos expansion (PCE), originally developed by Wiener [21], as a possible way out of this difficulty. Using PCE, Sobol’ indices can be calculated analytically.

Referring to the problem of ’equifinality’ of different acceptable models, Beven and Freer [22] developed the generalized likelihood uncertainty estimation (GLUE) methodology in which model simulations are ranked according to their performance. Interrelationships between parameters that lead to satisfactory results are implicitly represented by the respective subset of Monte Carlo sampling. In contrast to Bayesian Monte Carlo (BMC) [23], for instance, GLUE does not employ likelihood in a statistically rigorous sense. For a comparison of GLUE with formal Bayesian approaches, including also Markov Chain Monte Carlo (MCMC), see Vrugt et al. [24] or Camacho et al. [25], for instance. Ratto et al. [26] combined GLUE and GSA, replacing model output variability in GSA by the variability of an informal GLUE likelihood measure. Callies et al. [27] applied the GSA-GLUE approach to a model, which is similar to the model [28] the present case study refers to.

There exist numerous related works treating blackbox models with functional outputs with surrogates. Campbell et al. [29] use an adaptive basis of principal component analysis (PCA) to perform global sensitivity analysis. Pratola et al. [30] and Ranjan et al. [31] use GP regression for sequential model calibration in a Bayesian framework. Lebel et al. [32] model the likelihood function in an MCMC model calibration via a Gaussian process. Perrin [33] compares the use of a multi-output GP surrogate with a Kronecker structure to an adaptive basis approach. Extensions presented here rely on the adaptive basis approach in principal components (Karhunen–Loéve expansion or functional PCA) to reduce the dimension of the functional output, while modeling the map from inputs to weights in this basis via GP regression.

## 3. Case Study: Modeling Chlorophyll a Concentrations at Geesthacht Weir

### 3.1. General Background

**Figure 1:**
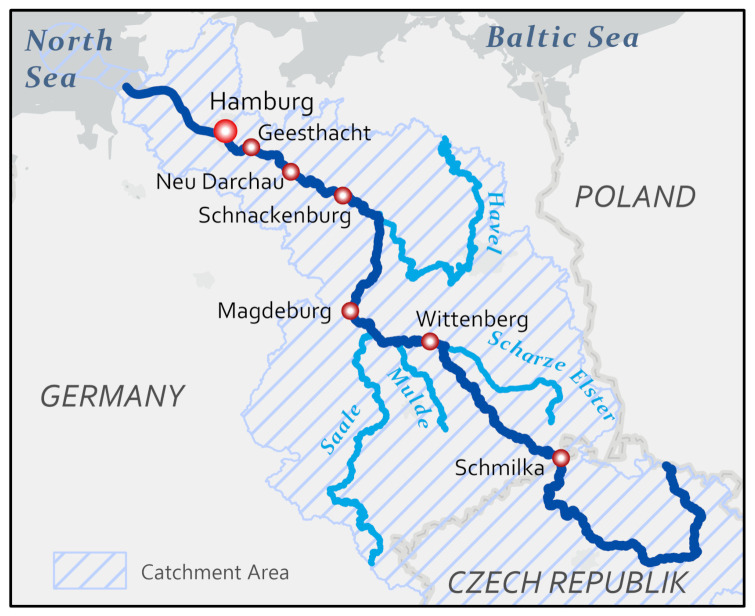
The Elbe River with station Geesthacht where the chlorophyll *a* and silica observations under study were taken. Some aspects of model forcing were obtained from stations Neu Darchau (river discharge), Schnackenburg (temperatures in 1997) and Schmilka (silica). The map also indicates the four most important tributaries.

Weir Geesthacht, located on the Elbe River (Elbe km 586) some 40 km upstream of the city of Hamburg (Figure 1), separates the riverine part of the Elbe River from its estuary issuing into the North Sea. Quasi-continuous observations of several parameters (see [28]), obtained from an automated flow-through unit operated at the weir by the former GKSS Research Center Geesthacht (now Helmholtz-Zentrum Hereon), are available for the years 1997–2001. Here, we focus on concentrations of chlorophyll *a* (obtained from observed fluorescence, using calibration based on high-performance liquid chromatography (HPLC)) and silica, observed during March-October. Chlorophyll *a* data were collected quasi-continuously, silica on an hourly basis.

Our case study on the use of Bayesian methods will be based on a very simple water quality model published previously. Considering no other algal species than diatoms, the model simulates chlorophyll *a* time series at Geesthacht Weir as a function of few environmental parameters. We do not consider other observations available at Geesthacht Weir (oxygen, pH, turbidity, nutrients) as our intention is not to improve the model. We rather wish to demonstrate the presence of overparameterization in even this simple model and how Bayesian methods can tackle this problem and make users aware of it.

According to Karrasch et al. [34], diatoms dominate algae biomass in the Elbe River. Our diatom-based model fits chlorophyll *a* observations quite well, despite its simplicity. For the Rhine river, de Ruyter van Steveninck et al. [35] identified a potential silica limitation effect during an experiment in 1990. Generally, it is very difficult to identify those among different biological processes that really control observed phytoplankton growth. Numerical models provide a means to at least formalize different hypotheses, estimate their consequences and compare them with observational evidence.

The complexity of the example model described below is much lower than that of most other mechanistic models trying to resolve processes in more detail, e.g., [36]. A key feature of the simple model is its ability (depending on how parameter values are set) to explain sporadic sharp decreases in chlorophyll *a* concentrations in terms of diatoms suffering from lack of silica [28]. At station Geesthacht, very low silica concentrations (below 0.1 mg Si/L) are observed during summer. However, the model also offers temperature dependent grazing rates as a potential alternative mechanism. Hardenbicker et al. [37] report an experimental study on major differences between the plankton dynamics in the two rivers Rhine and Elbe. They try to substantiate the hypothesis that much lower phytoplankton densities in the Rhine than in the Elbe River might be due to grazing by invasive bivalves being more abundant in the Rhine than in the Elbe River. Similarly, Waylett et al. [38] argue that between-year differences in grazing are likely to explain interannual variability of phytoplankton loss observed in the upper Thames. Although the temperature dependent loss rate assumed in our model is very simplistic compared to real world conditions, model calibration can nevertheless be hoped to indicate how distinguishable such temperature dependent mechanisms are from silica related effects.

### 3.2. Lagrangian Model Concept

For our case study, we revive a simple model originally introduced by Callies et al. [27] and even further simplified (neglect of all algae species other than diatoms, assumption of constant maximum growth rates of diatoms, no shading by mineral compounds) by Scharfe et al. [28]. Using a Lagrangian model concept originally suggested by Schroeder [39], fluid parcels are released at Schmilka close to the Czech–German border (Figure 1). These fluid parcels are then assumed to travel downstream until they reach Geesthacht Weir. During its journey, each parcel is treated like a biological reactor exposed to time dependent external forcing. Ideally, simulations would be complemented by Lagrangian sampling campaigns, trying to follow water parcels during their transport, e.g., [35,37]. However, the data available for this study provide observations just at the drift paths’ end points so that concentrations simulated on a fluid parcel’s arrival at Geesthacht Weir will be contrasted with corresponding observations at that time. For each parcel travelling down the Elbe River, the following equation for chlorophyll *a* concentration $C_{\mathrm{chl}}$ is integrated in time *t*:

(1) $$\frac{dC_{\mathrm{chl}}}{dt}=\left[\mu (t)-\sigma (t)\right]C_{\mathrm{chl}}$$

Time dependent growth rate μ and loss rate σ will be further detailed in Section 3.3.

The potential for chlorophyll *a* development is assumed to depend on the amount of silica being available. Our simple model concept assumes that an initial reservoir of silica, $C_{\mathrm{Si}}\left(t_{0}\right)$, is continuously depleted due to assimilation of silica by algae. The following equation describes the evolution of silica concentration $C_{\mathrm{Si}}$ in a given fluid parcel,

(2) $$\frac{dC_{\mathrm{Si}}}{dt}=-\mu \left(t\right)f_{\mathrm{Si}}C_{\mathrm{chl}}$$

where parameter $f_{\mathrm{Si}}$ specifies the ratio of silica to chlorophyll *a* in algal biomass. Note that Equation (2) does not take into account any sources of silica, like releases from the sediment or inputs from tributaries. Following Scharfe et al. [28], we initialized Equation (2) with concentrations observed at station Schmilka near the Czech–German border (Elbe km 4, see Figure 1). This station marks the end of the upper reach of the river (about 370 km long) with a mean river discharge of about 310 m${}^{3}$/s (compared to about 730 m${}^{3}$/s at Geesthacht Weir, 580 km further downstream). Initial concentrations were constrained, however, by a minimum value of 2 mg Si/L.

Simulations for different times are completely independent amongst each other, any temporal coherence at Geesthacht Weir is brought about just by the fact that external forcing (light and temperature) will be the same for trajectories overlapping in time. Each fluid parcel is initialized with the same low chlorophyll *a* concentration (10 μg chl *a*/L), a value expected to be largely overwritten during the water parcel’s 580 km journey. What might contribute to the success of the very simple approach is that in nature there seem to be only small contributions of chlorophyll *a* from major tributaries of the river Elbe [37].

It must be stressed that our drift simulations take into account neither variations in river geometry nor any dispersion processes or supply from external sources. The only consequence of changing river discharge is a changing travel time, i.e., the time available for algae growth and loss processes. Travel times τ (usually less than 10 days) are estimated as function of discharge *Q*,

(3) $$\tau =\tau_{\mathrm{ref}}{\left(\frac{Q_{\mathrm{ref}}}{Q}\right)}^{1/3}$$

with reference values $Q_{\mathrm{ref}}=270\;m^{3}/s$ and $\tau_{\mathrm{ref}}=10$ days. This simple formula was found to reasonably agree with existing flow time data [40].

Simulations are scheduled in such a way that drift trajectories arrive once a day at noon. For a more detailed illustration of the Lagrangian approach, also showing examples of concentrations that develop within single fluid parcels, the reader is referred to the original paper of Scharfe et al. [28].

### 3.3. Parameterizations Used in the Model

In Equation (1), both μ and σ depend on environmental conditions and therefore vary with time *t*. The value of μ(t) results as the triple product of a constant maximum growth rate $\mu_{0}$, a light dependent limitation factor $F_{\mathrm{light}}\left(t\right)$ parameterizing restricted growth efficiency under unfavorable light conditions and another limitation factor $F_{\mathrm{Si}}\left(t\right)$ parameterizing detrimental effects of possibly limited availability of silica:

(4) $$\mu \left(t\right)=\mu_{0}F_{\mathrm{light}}\left(t\right)F_{\mathrm{Si}}\left(t\right)$$

Both $F_{\mathrm{light}}\left(t\right)$ and $F_{\mathrm{Si}}\left(t\right)$ can assume values between 0 and 1.

According to Beer’s law, radiation intensity I(t) at the water surface implies a radiation intensity $I\left(t\right)e^{-\lambda (t)z}$ at water depth *z*. Here, coefficient λ(t) is used to parameterize light attenuation due to so-called algal self-shading, assumed to be proportional to chlorophyll *a* concentration,

(5) $$\lambda \left(t\right)=\lambda_{S}C_{\mathrm{chl}}\left(t\right)$$

with some constant factor $\lambda_{S}$. Our simple model does not explicitly resolve the water depth coordinate *z*, so that the time dependent light limitation factor $F_{\mathrm{light}}$ in Equation (4) is obtained by vertical averaging over water depth *D*. The following formula,

(6) $$F_{\mathrm{light}}\left(t\right)=\frac{1}{D}\int_{0}^{D}\frac{I\left(t\right)\exp^{-\lambda (t)z}}{\sqrt{K_{\mathrm{light}}^{2}+I^{2}\left(t\right)\exp^{-2\lambda (t)z}}}dz$$

is based on the ‘Smith formula’ [41]. If light intensity equals the half-saturation constant $K_{\mathrm{light}}$, algal growth rate will assume 71% of its maximum possible value. Global radiation on an hourly basis was taken from GKSS Research Center located in close vicinity to the weir.

During trajectory calculation, water depth *D* is treated as a constant. For each individual trajectory, however, the value of *D* is adjusted to the water discharge observed at station Neu Darchau (about 50 km upstream of Geesthacht) at the time when this trajectory reaches Geesthacht Weir. A polynomial formula well reproduces an empirical relationship between discharge and water depth, although slightly enhancing small values of *D* (see [28], their Figure 3).

A half-saturation constant $K_{\mathrm{Si}}$ is introduced to specify limitation factor $F_{\mathrm{Si}}$ in Equation (4):

(7) $$F_{\mathrm{Si}}\left(t\right)=\frac{C_{\mathrm{Si}}\left(t\right)}{K_{\mathrm{Si}}+C_{\mathrm{Si}}\left(t\right)}$$

Parameter $K_{\mathrm{Si}}$ is set to the fixed value of 0.1 mg Si/L. A silica concentration equal to this value will imply a 50% reduction in growth rate μ in Equation (4).

Scharfe et al. [28] identified in each of the five years, 1997–2001, a short period when the model consistently failed to reproduce a fast increase in chlorophyll *a* concentrations after a late spring chlorophyll *a* minimum. This model deficiency could not be fixed by any parameter adjustments, which suggests relevance of certain processes that are lacking in the present model framework (e.g., dominance of algae other than diatoms). The authors therefore decided to modify the model in such a way that in each year, assimilation of silica is abandoned during a 1–2 week period (see [28], Figure 11 therein). In this study, we adopt this approach to prevent the large short-term discrepancies dominating the overall model evaluation. In all time series shown in this paper, the special periods will be highlighted. The workaround was chosen because any extension of the model would be beyond the scope of the present study.

Loss rate σ (including also respiration) in Equation (1) is assumed temperature dependent only if temperature *T* exceeds 20 ${}^{\circ }$C, otherwise it is set constant:

(8) $$\sigma \left(t\right)=\left\{\begin{array}{cc} \sigma_{0}\; & \mathrm{for}\;T<20{\;}^{\circ }C\\ \sigma_{0}a^{T\left(t\right)-20{\;}^{\circ }C}\; & \mathrm{for}\;T\geq 20{\;}^{\circ }C \end{array}\right.$$

The interpretation of coefficient *a* being greater than one remains unspecific but could cover an increased zooplankton grazing rate, for instance. Evaluation of Equation (8) is based on 24 h means of water temperature at Geesthacht Weir. Only for the year 1997, data from station Schnackenburg (Elbe km 475, from the former ARGE ELBE; since 2010, part of the ‘Flussgebietsgemeinschaft Elbe–FGG Elbe’; https://www.fgg-elbe.de, accessed on 29 September 2021) had to be used to fill existing data gaps.

### 3.4. Parameters Selected for Calibration

From the above equations, we selected six parameters for this calibration study: maximum growth rate $\mu_{0}$, half-saturation constant $K_{\mathrm{light}}$, light attenuation constant $\lambda_{S}$, algal silica content $f_{\mathrm{Si}}$, loss rate $\sigma_{0}$ and coefficient *a* for loss rate temperature dependence above 20 ${}^{\circ }$C. These parameters cover all essential aspects of the model.

## 4. Methods of Bayesian Analysis and Complexity Reduction

Here, we provide an introductory overview of the Bayesian methods used to calibrate and analyze the above-described model. A more detailed introduction to Bayesian concepts and techniques can, e.g., be found in [7,8]. In addition, Gaussian graphical models and principal component analysis are briefly outlined, as well as tools for pre- and postprocessing.

### 4.1. Bayesian Inference

Model calibration means estimating a set of *M* model parameters $\theta =(\theta_{1},\theta_{2},\cdots ,\theta_{M})$ from observed data d. As mentioned in the introduction, both input and output of the Bayesian parameter estimation procedure are probability distributions in θ. Before considering d, we choose a *prior* distribution p(θ) that contains all previous knowledge independent of d (e.g., certain parameters are positive or limited by plausible physical boundaries). Even though procedural models usually yield deterministic output for a given parameter vector θ, observational data d are subject to measurement uncertainties or noise. The further away d is from the model prediction, the less likely the parameters θ are correct. Quantitatively, this is encoded in the *likelihood*p(d∣θ), being the conditional probability to observe d for given θ. Since the given information is observed data d rather than abstract parameters θ, our goal is to estimate the *posterior* distribution p(θ∣d) where the conditionality is flipped compared to the likelihood. For this purpose, one applies the chain rule of conditional probabilities,

(9) p(θ∩d)=p(θ∣d)p(d)=p(d∣θ)p(θ),

transformed to Bayes’ rule

(10) $$p\left(\theta |d\right)=\frac{p(d|\theta )p(\theta )}{p(d)}.$$

The probability

(11) $$p\left(d\right)=\int p\left(d|\theta \right)p\left(\theta \right)d^{M}\theta ,$$

to observe d at all for a given model over all possible parameter sets is called the *marginal likelihood* or *evidence*. While for parameter estimation in a single model, p(d) cancels out as a normalization factor, and it becomes important when comparing several models.

### 4.2. Markov Chain Monte Carlo (MCMC)

Markov Chain Monte Carlo (MCMC) [7,42,43,44] is a method to obtain access to unbiased samples from potentially high-dimensional probability distributions. These samples can then be used to compute quantities of interest such as parameter means and variances. Here, we are interested in samples distributed according to the posterior p(θ∣d) for the model parameters θ given the data d via Equation (10). The key idea underlying the MCMC approach is an iterative exploration of a target probability distribution such that the distribution of the samples asymptotically converges to it. In the commonly used Metropolis–Hastings algorithm, proposal steps (i.e., changing the parameter values from θ to $\theta^{{}^{\prime }}$) which increase the probability are always accepted. If, however, the probability is reduced, then such a parameter step is only accepted with an acceptance probability $\alpha (\theta ,\theta^{{}^{\prime }})$, i.e.,

(12) $$\alpha \left(\theta ,\theta^{{}^{\prime }}\right)=\min\left(1,\frac{p\left(\theta^{{}^{\prime }}|d\right)}{p\left(\theta |d\right)}\right)$$

for symmetric proposal functions ([43], chapter 15.8). If a proposed step is not accepted, then the old parameter vector θ is added again to the chain of sampled parameter values, otherwise the system state is set to the new value $\theta^{{}^{\prime }}$. Under some weak technical conditions (such as ergodicity, detailed balance), the distribution of this chain converges to the desired distribution p(θ∣d) [45].

Let now θ denote the vector of M=6 model parameters $\theta_{k}$ (see Table 1) we wish to calibrate based on existing observations. Let $d_{t}$ be a time series with $N_{t}$ observations of chlorophyll *a* depending on time *t*, and $f_{t}\left(\theta \right)$ the corresponding output of one chlorophyll *a* simulation. Note that $N_{t}$ is not the number of samples, but each sample is a time series of length $N_{t}$ that is compared to the observed time series. Model calibration is based on the cost function

(13) $$J\left(\theta \right)=\frac{1}{2N_{t}}\sum_{i=1}^{N_{t}}\frac{{\left[f_{i}\left(\theta \right)-d_{i}\right]}^{2}}{\sigma_{\mathrm{chl}}^{2}}-\sum_{k=1}^{M}\ln p\left(\theta_{k}\right)$$

with an assumed standard deviation $\sigma_{\mathrm{chl}}$ of the observational error, assumed as a Gaussian random variable. Instead of just minimizing *J*, we use it as the negative log-likelihood in a Gaussian error model for probabilistic inference. The MCMC search algorithm should not explore unrealistically large values for parameters or parameter combinations insufficiently controlled by observations. To realize this while not being overly restrictive, for each parameter $\theta_{k}$, a heavy-tailed Cauchy distribution is introduced as a prior:

(14) $$p\left(\theta_{k}\right)=\frac{2}{\pi }\frac{b_{k}}{b_{k}^{2}+\theta_{k}^{2}}.$$

All parameters $\theta_{k}$ considered in this study are constrained to positive values. Thus, we truncate the prior to vanish for values $\theta_{k}<0$. Scaling ensures that $\int_{0}^{\infty }p\left(\theta_{k}\right)d\theta_{k}=1$. Coefficients $b_{k}$ in Equation (14) control the width of the probability distribution for each parameter $\theta_{k}$. This width is fixed by specifying values $\theta_{k}^{★}$ that to be exceeded should be quite unlikely (see Table 1). Values for $b_{k}$ are chosen such that the probability to find $\theta_{k}<\theta_{k}^{★}$ is close to one. This probability is given by the respective value of the cumulative distribution function

(15) $$P^{★}=\int_{0}^{\theta_{k}^{★}}p\left(\theta_{k}\right)d\theta_{k}.$$

With Equation (14) and $\int {\left(b_{k}^{2}+\theta_{k}^{2}\right)}^{-1}d\theta_{k}=\arctan\left(\theta_{k}/b_{k}\right)/b_{k}$, coefficients $b_{k}$ satisfying Equation (15) can then be calculated as:

(16) $$b_{k}=\theta_{k}^{★}\tan^{-1}\left(\frac{\pi }{2}P^{★}\right).$$

In the following, we assume a value of $P^{★}=0.9$.

In the MCMC algorithm, positivity of parameters $\theta_{k}$ is warranted by flipping negative proposed parameter test values into the positive range by taking absolute values. This approach reproduces the truncated prior and maintains the detailed balance necessary for the convergence of MCMC [8]. Freni and Mannina [46] studied the implications of choosing prior distributions on uncertainty analysis. Here, we sought to keep prior constraints as weak as possible. A more rigorous alternative to do so would be the use of the maximum entropy principle to minimize the amount of prior information. This is not performed here for simplicity, since we expect that the number of samples is sufficiently large to make the influence of the exact form of the prior on the posterior negligible for practical purposes.

### 4.3. Graphical Modeling

Graphical models can be used to highlight key interrelationships between parameters, discarding dependencies of minor importance. Data are represented in terms of nodes (or vertices) for each variable and a number of edges connecting them. Edges in undirected Gaussian graphical models (e.g., [47,48]) represent partial correlations (i.e., correlations between pairs of variables when all other variables are held constant). By contrast, directed edges in Bayesian networks (e.g., [9,10]) represent conditional probability distributions approximated by tables for response variables (child nodes) given the values of all explanatory variables (parent nodes) from which edges are pointing into them.

#### 4.3.1. Gaussian Graphical Models (GGMs)

Undirected edges in a GGM represent non-zero pairwise partial correlations conditioned by all the rest of the variables. Covariance selection developed by Dempster [49] provides a general framework to assess whether or not the set of constraints displayed by a GGM contradicts observations. See Whittaker [48] for a comprehensive presentation of the concept. More recent developments of variational methods are described in Jordan [50]. Callies [51] and Callies and Scharfe [52] applied graphical modeling for analyzing interaction structures from water quality observations; Taeb et al. [53] used it to characterize dependencies among water reservoirs. The specific aspect in this study is that we do not apply the method to observed variables but rather to a set of parameters in a process-oriented simulation model that were calibrated in order to adjust model output (time series of one single variable) to its observed counterpart.

The basic idea of graphical Gaussian modeling is to modify a sample correlation matrix S within the limits of observational uncertainty in such a way that small partial correlations are replaced by zero values. The partial correlation matrix $S_{p}$ is closely related to the precision matrix $S^{-1}$ and zero valued elements of the two matrices coincide, see [48]. Among all correlation matrices that satisfy the constraints of a given GGM, some matrix V will fit the data best. The difference between the log-likelihoods of sample correlation matrix S and V provides an entropy type measure of the amount of information in the data against the interaction structure hypothesized by the graph *G*. The deviance ${\mathrm{dev}}_{S}\left(G\right)$ is defined as twice this difference of log-likelihoods or twice the sample size *N* times the Kullback–Leibler information divergence between two jointly normal distributions, assuming that their means are equal [54]. Specific properties of V imply that the deviance assumes the following simplified form [47]:

(17) $${\mathrm{dev}}_{S}\left(G\right)=N\ln\left(\frac{\left|V\right|}{\left|S\right|}\right)$$

If data are normally distributed, the deviance has an asymptotic $\chi^{2}$ distribution with the degrees of freedom given by the number of edges missing in the graph [48].

With Equation (17), it is straightforward to evaluate the effects of either excluding another edge from the graph (edge excluding deviance, EED) or re-establishing an edge previously removed (edge inclusion deviance, EID). In this study, we will not rely on statistical significance of graph simplification. Due to the very large number of samples, i.e., the number of underlying successful Monte Carlo simulations ($N=10^{6}$), formal statistical significance will always be satisfied. Strict statistical testing for model parameter interactions would also be inconsistent with the fact that even very detailed process-oriented models necessarily comprise substantial simplifications and parameterization of much more detailed natural processes. In the light of this unavoidable incorrectness of any process-oriented model, we try to characterize the model’s key interaction structure by stopping graph simplification at a point when the smallest EED in the simplified graph *G* is clearly larger than the largest EID among the EIDs of all edges discarded previously. It must be noted that this graph simplification is a manual and to some extent subjective procedure, complicated by the fact that removal or establishment of an edge generally can affect the relevance of all other edges.

#### 4.3.2. Bayesian Networks (BNs)

Contrary to Gaussian graphical models, Bayesian networks are directed acyclic graphs (DAGs) [9,10]. Nodes of the BN represent random variables with usually discrete states, often (as in our case) obtained by binning a continuous variable into a certain number of categories. For each node, a table specifies the conditional probabilities for its states, given all possible combinations of states of the node’s ancestors according to the DAG. The joint distribution for *N* variables $X_{N}$ is then given as a product of conditional probabilities,

(18) $$P\left(X_{1},...,X_{N}\right)=\prod_{X_{i}\ldots \left\{X_{i},\ldots ,X_{N}\right\}}P\left(X_{i}|\mathrm{Pa}\left(X_{i}\right)\right)$$

where $\mathrm{Pa}(X_{i})$ denotes the set of all parent nodes of node $X_{i}$. For root nodes without parents (applies to at least one node in a DAG), the conditional probability $P(X_{i}|Pa\left(X_{i}\right))$ is replaced by the simple prior distribution $P(X_{i})$. Structuring a BN in terms of parent and child nodes can often be related to the concept of causality [11,12]. However, for nodes representing parameters of a process model, such an interpretation is not applicable.

Interactive BN software provides a convenient tool to explore parameter dependences empirically. However, depending on how many state categories are used for each parameter, the maximum number of M−1 parent nodes (with *M* denoting the number of parameters) can be a serious limitation for the application of BN software. That is why, for saturated graphs (with all edges being maintained), specification of conditional marginal distributions by sampling directly from the data (see Section 4.8) may be preferable to specification of conditional probability tables. Omission of edges from the graph can much reduce the problem of dimensionality. For nearly multinormal distributions, graphical Gaussian modeling may guide such simplification of a BN.

### 4.4. Gaussian Process Regression and Bayesian Global Optimization

Gaussian process regression [55,56,57] is a commonly used tool to construct flexible non-parameteric surrogates. Based on observed outputs $f(x_{k})$ at training points $x_{k}$ and a covariance function $k(x,x^{\prime })$, the GP regressor predicts a Gaussian posterior distribution at any point $x^{*}$. For a single prediction $f(x^{*})$, expected value and variance of this distribution are given by

(19) $$\begin{array}{cc} \bar{f}\left(x^{*}\right) & =m\left(x^{*}\right)+K^{*}{\left(K+\sigma_{n}I\right)}^{-1}d, \end{array}$$

(20) $$\begin{array}{cc} \mathrm{var}[f\left(x^{*}\right)] & =K^{**}-K^{*}{\left(K+\sigma_{n}I\right)}^{-1}K^{*T}, \end{array}$$

where $m(x^{*})$ is the mean model, the covariance matrix K contains entries $K_{ij}=k\left(x_{i},x_{j}\right)$ based on the training set, $K_{i}^{*}\left(x^{*},x_{i}\right)$ are entries of a row vector and $K^{**}=k\left(x^{*},x^{*}\right)$ is a scalar. The unit matrix I is added with the noise covariance $\sigma_{n}$ that regularizes the problem and is usually estimated in an optimization loop together with other kernel hyperparameters.

Such a surrogate with uncertainty information can be used for Bayesian global optimization [58,59,60] of the log-posterior as a cost function. Here, we apply this method to reach the vicinity of the posterior’s mode before sampling. As an acquisition function, we use the expected improvement (see, e.g., [59]) at a newly observed location $x^{*}$ given existing training data d,

(21) $$\begin{array}{cc} a_{\mathrm{EI}}\left(x^{★}\right) & =E[\max\left(0,\bar{f}\left(x^{*}\right)-\hat{f}\right)|x^{*},d]\\ \; & =\left(\bar{f}\left(x^{*}\right)-\hat{f}\right)\Phi \left(\hat{f};\bar{f}\left(x^{*}\right),\mathrm{var}\left[f\left(x^{*}\right)\right]\right)+\mathrm{var}\left[f\left(x^{*}\right)\right]N\left(\hat{f};\bar{f}\left(x^{*}\right),\mathrm{var}\left[f\left(x^{*}\right)\right]\right), \end{array}$$

where $\hat{f}$ is the optimum value for f(x) observed so far. Due to the non-linear transformation from the functional blackbox output to the value of the cost function, it is more convenient to realize Bayesian optimization with a direct GP surrogate of the cost function that is constructed in addition to the surrogate for the functional output for the KL expansion coefficients described below.

### 4.5. Linear Dimension Reduction via Principal Components

Formally, the blackbox output for given input x can be a function f(t)…H in an infinite-dimensional Hilbert space (though sampled at a finite number of points in practice). Linear dimension reduction in such a space means finding the optimum set of basis functions $\varphi_{k}\left(t\right)$ that spans the output space f(t;x) for any input **x** given to the blackbox. The reduced model of order *r* is then given by

(22) $$f\left(t;x\right)\approx \sum_{k=1}^{r}z_{k}\left(x\right)\varphi_{k}\left(t\right).$$

This approach is known as the Karhunen–Loéve (KL) expansion [61], in case f(t;x) are interpreted as realizations of a random process, or as the functional principal component analysis (FPCA) [62]. For our application, this distinction does not matter. The KL expansion boils down to solving a regression problem in the non-orthogonal basis of *N* observed realizations to represent new observations. Then an eigenvalue problem is solved to invert the N×N collocation matrix A with entries

(23) $$A_{ij}=\langle f\left(t;x_{i}\right),f\left(t;x_{j}\right)\rangle .$$

Here, the inner product in Hilbert spaces and its approximation for a finite set of support points is given by

(24) $$\langle u,v\rangle =\int_{\Omega }u\left(t\right)v\left(t\right)\;dt\approx \frac{1}{N_{t}}\sum_{k=1}^{N_{t}}u\left(t_{k}\right)v\left(t_{k}\right).$$

If $N_{t}\gg N$ (many support points, few samples), solving the eigenvalue problem of the collocation matrix A is more efficient than the dual one of the covariance matrix C with $C_{ij}=\sum_{k}f\left(t_{i},x_{k}\right)f\left(t_{j},x_{k}\right)$ in the usual PCA (see [56] for their equivalence via the singular value decomposition of $Y_{ij}=f\left(t_{i},x_{j}\right)$). The question at which *r* to truncate the eigenspectrum in (22) depends on the desired accuracy in the output that is briefly analyzed in the following paragraph.

Here, we justify why we can assume an $L_{2}$ truncation error of the order of the ratio $\lambda_{r}/\lambda_{1}$ between the smallest eigenvalue considered in the approximation and the largest one. The truncated SVD can be shown to be the best linear approximation $A^{\left(r\right)}$ of lower rank *r* to an N×N matrix A in terms of the Frobenius norm ${\left||A|\right|}_{F}$ (see, e.g., [63]). Its value is simply computed from the $L_{2}$ norm of singular values,

(25) $${\left||A|\right|}_{F}={\left(\sum_{k=1}^{N}\sigma_{k}^{\;2}\right)}^{1/2},$$

where $\sigma_{k}^{\;2}=\lambda_{k}$ in case of real eigenvalues $\lambda_{k}$ of a positive semi-definite matrix as for the covariance or collocation matrix. The truncation error is given by

(26) $$\left|\right|A^{\left(r\right)}-A{\left|\right|}_{F}={\left(\sum_{k=r+1}^{N}\lambda_{k}\right)}^{1/2}.$$

The error estimate for the KL expansion uses this convenient property together with the fact that the Frobenius norm is compatible with the usual $L_{2}$ norm |x| of vectors y, i.e.,

(27) $$\left|Ay\right|\leq {\left||A|\right|}_{F}\left|y\right|.$$

Representing y via the first *r* eigenvalues of the collocation matrix yields a relative squared reconstruction error of

(28) $$|\left(A^{\left(r\right)}-A\right){y|}^{2}/{\left|y\right|}^{2}\leq \sum_{k=r+1}^{N}\lambda_{k}\leq \left(N-r\right)\lambda_{r}.$$

The last estimate is relatively crude if N≫r and the spectrum decays fast with the index variable *k*. If one assumes a decay rate α with

(29) $$\lambda_{k}\approx \lambda_{r}{\left(k-r\right)}^{-\alpha },$$

one obtains

(30) $$\sum_{k=r+1}^{N}\lambda_{k}\approx \sum_{k=r+1}^{\infty }\lambda_{r}{\left(k-r\right)}^{-\alpha }=\lambda_{r}\sum_{k=1}^{\infty }k^{-\alpha }=\lambda_{r}\zeta \left(\alpha \right),$$

where ζ is the Riemann zeta function. This function diverges for a spectral decay of order α=1 and reaches its asymptotic value ζ(∞)=1 relatively quickly for α≥2 (e.g., ζ(3)=1.2). The spectral decay rate α can be fitted in a log–log plot of $\lambda_{k}$ over index *k* and takes values between α=3 and 5 in our use case. The underlying assumptions are violated if the spectrum stagnates at a large number of constant eigenvalues for higher indices *k*.

### 4.6. Delayed Acceptance MCMC

Delayed acceptance MCMC builds on a fast surrogate for the posterior $\tilde{p}\left(\theta |d\right)$ to reject unlikely proposals early [13,14]. Following the usual Metropolis–Hastings algorithm, the probability to accept a new proposal $\theta^{*}$ in this first stage in the *n*-the step of the Markov chain is as usual,

(31) $${\tilde{P}}_{\mathrm{acc}}^{n}=\frac{\tilde{p}\left(\theta^{*}|d\right)}{\tilde{p}\left(\theta_{n-1}|d\right)}\frac{g(\theta_{n-1}|\theta^{*})}{g(\theta^{*}|\theta_{n-1})},$$

where *g* is a transition probability that has been suitably tuned during warmup. The true posterior p(θ|d) is only evaluated if the proposal ‘survives’ this first stage and enters the final acceptance probability

(32) $$P_{\mathrm{acc}}^{n}=\frac{p(\theta^{*}|d)}{p(\theta_{n-1}|d)}\frac{\tilde{p}\left(\theta_{n-1}|d\right)}{\tilde{p}\left(\theta^{*}|d\right)}.$$

Actual computation is, as usual, performed in the logarithmic space with cost function

(33) ℓ(θ|d)≡−logp(θ|d).

If this function is fixed, it is most convenient to just directly build a surrogate $\tilde{\ell }\left(\theta |d\right)$ for the scalar log-posterior (cost) function y=ℓ(θ|d) depending on x=θ including the corresponding prior. Below, we describe an alternative approach that models the full functional output instead.

### 4.7. Bayesian Hierarchical Models and Fractional Norms

One application of modeling the full functional output instead of only the cost function is the existence of additional distribution parameters ζ in the likelihood besides the original model inputs θ. Such dependencies appear within Bayesian hierarchical models [64], where ζ are again subject to a certain (prior) distribution with possibly further levels of hyperparameters. There are essentially two ways to construct a surrogate with support for additional parameters ζ: Building a surrogate for the cost function that adds ζ as independent variables or constructing a surrogate with functional output for $f_{k}\left(\theta \right)$ and keeping the dependencies on ζ exact. Here, we focus on the latter and apply this surrogate within delayed acceptance MCMC with both θ and ζ as tunable parameters.

As an example we use a more general noise model than the usual Gaussian likelihood that builds on arbitrary $\ell^{\zeta }$ norms [65,66,67] with real-valued ζ not fixed while traversing the Markov chain. We allow members of the exponential family for observational noise and specify only its scale but keep ζ as a free parameter. Namely, we model the likelihood for observing d in the output as

(34) $$p\left(d|\theta ,\zeta \right)=\frac{1}{2\sqrt{2}\sigma \;\Gamma \left(1+\zeta^{-1}\right)}e^{-\ell (d;\theta ,\zeta )},$$

with the normalized $\ell^{\zeta }$ norm to the power of ζ,

(35) $$\ell \left(d;\theta ,\zeta \right)\equiv \frac{1}{N_{t}}\sum_{i=1}^{N_{t}}{\left|\frac{f_{i}\left(\theta \right)-d_{i}}{\sqrt{2}\sigma }\right|}^{\zeta }$$

as the loss function between observed data $d_{i}$ and blackbox model $f_{i}\left(\theta \right)$. Choosing the usual $L_{2}$ norm leads to a Gaussian likelihood for the noise model, whereas using the $L_{1}$ norm means Laplacian noise. To maintain the relative scale when varying ζ, it is important to add the term $\log\Gamma (1+\zeta^{-1})$ from (34) to the negative log-likelihood. In the following use cases, we are going to compare the cases of fixed and variable ζ.

### 4.8. Pre- and Postprocessing

For analyzing measured data and the posterior distribution of model parameters, two techniques have been implemented in the interactive proFit [68] toolkit for probabilistic reduced order model fitting, using GPflow [69,70] and GPy [71] backends and visualization via Plotly/Dash. On the one hand, this concerns the estimation of noise in measured time-series data in absence of a parameterized model. On the other hand, the analysis of the posterior distribution of the calibrated parameters is facilitated by on-the-fly visualization of conditional marginal distributions.

In order to introduce a scale for the tolerated deviation in the MCMC calibration of model parameters, the random noise in the measured time-series data d has to be known or, as in the present case, estimated. For this purpose, a fairly general Gaussian process (GP) regression [57] with a squared-exponential kernel is applied to the data. Characteristic timescale and random noise are left as free parameters and optimized to their maximum-likelihood values based on the data. This yields a decomposition of a kernel-smoothed representation of the original data plus a Gaussian noise term $\sigma_{n}$. In order for this estimate to be valid, the random error must be sufficiently close to a normal distribution and the characteristic timescale should not vary over time.

An alternative way to estimate noise has been evaluated by keeping observational noise as a free parameter and inferring its value during MCMC sampling. This path has been abandoned, as it yields to an overestimation of noise in the present case. The reason is the following. In contrast to the GP regression, no combination of model parameters can eliminate all systematic deviations from the observed data. Inference with the present Gaussian likelihood model incorrectly identifies these deviations with noise and expands the confidence bands to enforce a match between model and data. In contrast, using the estimated noise from an empirical ‘perfect’ fit via a GP infers noise and confidence bands mainly from the data alone, without implicitly assuming correctness of the model.

For *M* parameters, the marginal distribution of the posterior for each model parameters $\theta_{k}$, given measured data d is given by

(36) $$p\left(\theta_{k}|d\right)=\int_{-\infty }^{\infty }p\left(\theta |d\right)\;d^{M-1}\theta_{i\neq k}.$$

Here, these marginal distributions $p(\theta_{k}|d)$ are approximately computed by taking sums of MCMC data inside histogram intervals. Similar to software for BNs, proFit allows to interactively restrict values of certain parameters to intervals $\left(\theta_{l}^{A},\theta_{l}^{B}\right)$ of a certain histogram bar and observe the influence on conditional marginal distributions

(37) $$p\left(\theta_{k}|d,\theta_{l}^{A,B}\right)=\frac{p(\theta_{k},\theta_{l}^{A,B}|d)}{p(\theta_{l}^{A,B}|d)}=\frac{\int_{-\infty }^{\infty }\int_{\theta_{l}^{A}}^{\theta_{l}^{B}}p\left(\theta |d\right)\;d\theta_{l}\;d^{M-2}\theta_{i\neq k,l}}{\int_{-\infty }^{\infty }\int_{\theta_{l}^{A}}^{\theta_{l}^{B}}p\left(\theta |d\right)\;d\theta_{l}\;d^{M-1}\theta_{i\neq l}}$$

of each parameter. This enables a fast exploration in parameter spaces that are too high-dimensional to be visualized directly.

## 5. Results

### 5.1. MCMC Sampling

Based on the cost function defined in Equation (13), MCMC was used to explore the posterior joint distribution of parameters θ. An observational error (standard deviation) of $\sigma_{\mathrm{chl}}=5\;\mu$g chl/L has been determined via the maximum-likelihood estimate of the noise term from a Gaussian process regression as described in Section 4.4. A total of 1000 parallel chains were calculated with 1000 iterations each, starting at randomized locations in parameter space. For each chain, 5 warm-up trajectories with 500 iterations were calculated. To reduce the computational burden, chlorophyll *a* concentrations at Geethacht Weir were simulated for just every third day. Acceptance rates close to 35% for all parameters indicated a reasonable choice of the individual proposal step size of the MCMC algorithm [7,72]. The distance of the Gelman–Rubin statistic *R* [73] to the asymptotic value of 1 was found to be less than $10^{-3}$ for the computed chains, thus yielding no indication for an insufficient sampling.

**Figure 2:**
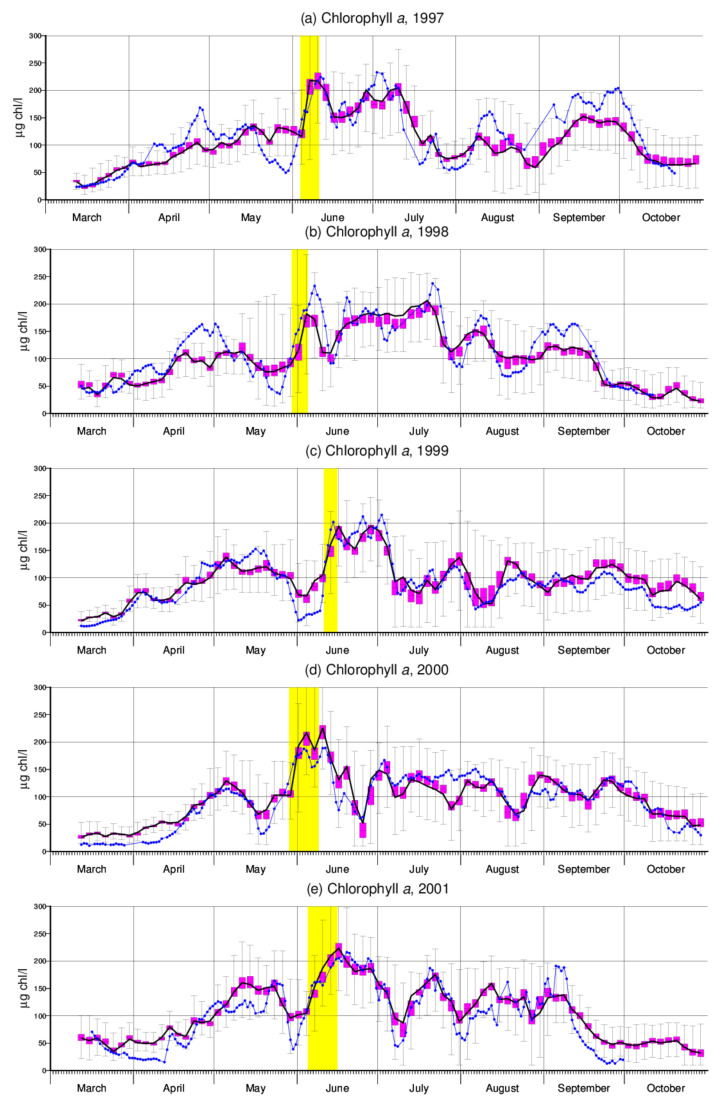
Chlorophyll *a* observations (blue) and corresponding simulations optimized to reproduce chlorophyll *a* observations in the five year period 1997–2001 shown in subplots (**a**–**e**). Black lines represent the simulation for which the cost function (Equation (13)) with $\sigma_{\mathrm{chl}}$ = 5 μg chl/L assumes a minimum value. Box plots represent the spread among simulations based on the $10^{6}$ feasible parameter sets obtained from MCMC. Yellow bars indicate periods during which the model assimilation of silica was abandoned (see Section 3.3).

Simulations based on $10^{6}$ feasible parameter combinations obtained from MCMC are summarized in Figure 2. Each parameter combination was assessed based on model performance during the five-year period 1997–2001 rather than during individual years. Black lines represent simulations based on those parameters for which the five-year cost function takes its minimum. Observations (daily mean values) are shown in blue. Only every third observation has a simulated counterpart.

In Figure 2, the spread in model outputs arising from parameter uncertainties is represented by the means of box plots. A total 50% of simulations are close to each other (magenta colored boxes). However, there is a surprisingly large spread between the extremes for each day. One may ask how this goes together with the positive evaluation as a reasonable simulation.

**Figure 3:**
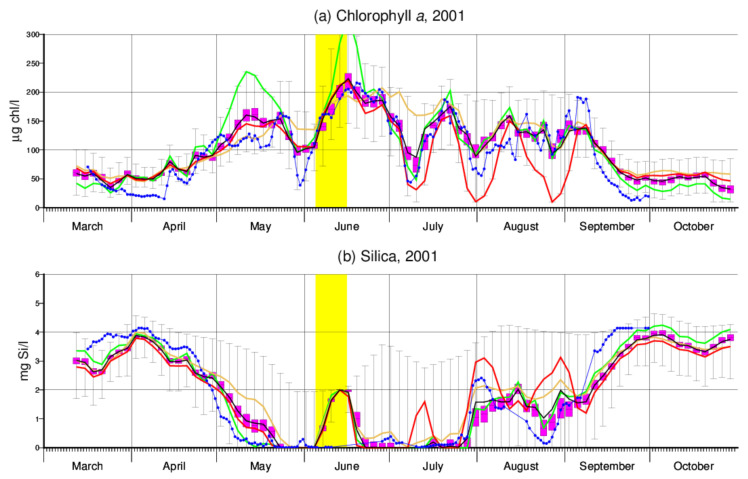
(**a**) Data (blue) and best fitting simulation (black) of chlorophyll *a* including uncertainties (box plots), copied from Figure 2e. Additionally, three simulations are shown that produce the maximum simulation at 11 May (green), 10 July (brown) or the minimum value on 31 July (red). The underlying parameter sets are listed in Table 2. (**b**) Corresponding data, simulations and simulation uncertainties for SiO${}_{2}$. Yellow bars indicate periods during which the model assimilation of silica was abandoned (see Section 3.3).

First, larger deviations may occur in simulations for specific years because model performance was evaluated for the five-year period in total. Second, the relevance of different parameters for model output depends on environmental conditions (e.g., temperature, availability of silica) at the time of interest, so that particularly large values of some parameters may lead to large anomalies at certain times while being of minor importance during other, probably longer, periods. To illustrate this effect, Figure 3a combines the optimum simulation for 2001 with three other simulations that produce the most extreme chlorophyll *a* values for 11 May, 10 July and 31 July, respectively. Table 2 compares the parameter values these three simulations are based on with the optimum (i.e., minimum cost function) simulation as a reference.

In Figure 3a, the green curve deviates from the others in that it shows particularly large peak values in May and June. This might be explained by a large maximum growth rate $\mu_{0}$ in combination with a large (compared to the reference) half-saturation constant $K_{\mathrm{light}}$ (Table 2). The latter assumption partly compensates for the large $\mu_{0}$ but at the same time makes growth rate μ more sensitive to variable light conditions (see Equation (6)). The brown curve to some extent follows an opposite approach, which results in a rather smooth simulation. At the end of July and August, it is then the red simulation that much overestimates two minima of chlorophyll *a*. This simulation is based on a very large parameter *a* (Table 2) which governs temperature dependence of algae loss rates above the 20 ${}^{\circ }$C threshold. The pronounced dips coincide with short periods of high temperatures ([28], Figure 9g therein).

Although algal silica content $f_{\mathrm{Si}}$ underlying the red curve in Figure 3a is the highest among the three example simulations (Table 2), the very low chlorophyll *a* concentrations imply low consumption of silica and therefore coincide with peak concentrations of this nutrient (Figure 3b). The upper bounds of silica ranges indicated in Figure 3b are more or less identical with concentrations prescribed at Schmilka where trajectories start (in the first half of June, the imposed lower bound of 2 mg Si/L can be noticed) and arise from zero consumption of silica. It is interesting to see that generally silica simulations look quite reasonable, although they were not used for model calibration. In fact, it turned out that their inclusion did not much affect the overall outcome of model calibration (not shown). Note that the simulated unrealistic increase in silica in June is due to the abandoning of silica consumption during the short period highlighted in yellow.

### 5.2. Principal Component Analysis of Feasible Parameter Combinations

**Figure 4:**
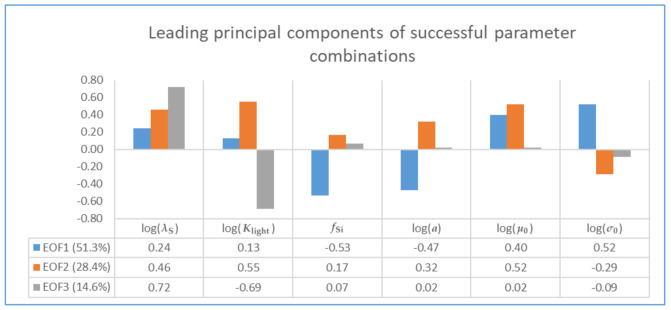
Principal component analysis applied to feasible parameter combinations obtained for the period 1997–2001. The graph shows three leading empirical orthogonal functions (EOFs) with corresponding PCs jointly accounting for approximately 94% of total parameter variability.

Posterior parameter dependences greatly influence model behavior, but their effects are not easily recognized in higher dimensions. Conventional principal component analysis of the parameter correlation matrix may be applied. Scaling is needed to remove different physical dimensions. If the six selected parameters in our study were strictly independent from each other, each of them (and also each principal component) would contribute 16.7% of overall parameter variability. It turns out, however, that already the first two principal components (PCs) describe 80% of overall parameter variation (see Figure 4). The spectrum of eigenvalues $\lambda_{k}$ can be used to estimate the statistical degree of freedom (dof) in parameter space [74]:

(38) $$\mathrm{dof}=\frac{M^{2}}{\sum_{k=1}^{M}\lambda_{k}^{2}}$$

**Figure 5:**
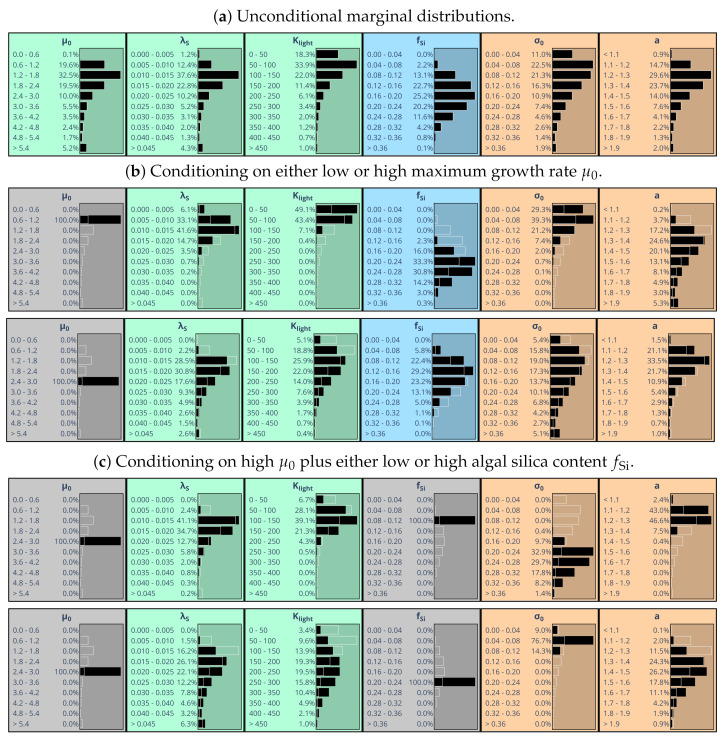
Each line combines 6 histograms that represent posterior marginal distributions of calibrated parameters (black bars). Background colors are used for grouping parameters into those related to algal growth (green), silica content (blue) and algal loss (brown). Grey colored histograms indicate that specific evidence regarding the respective parameter has been entered. To ease comparison, white contours in conditional distributions reproduce the unconditional distributions.

Here, we achieve dof = 2.7 for logarithmized (except $f_{\mathrm{Si}}$) data in M=6 dimensions. The logarithm was applied as five marginal distributions showed tails toward large values (see Section 5.3, Figure 5a). Empirical orthogonal functions (EOFs) describe the structure of parameter covariation underlying each mode of variability [75]. According to Figure 4, the two leading EOFs do not suggest a grouping of parameters or separation between different processes. Only the third EOF (explaining 13.8% of variance) clearly focuses on an interplay between parameters $\lambda_{S}$ and $K_{\mathrm{light}}$ (see Equations (5) and (6)).

### 5.3. Exploring Conditional Marginal Distributions

Discrete marginal parameter distributions, with continuous values of each parameter being binned into 10 classes, are shown in Figure 5a. Different colors were used to better distinguish between parameters related to different processes. Most distributions have tails toward large values. The only parameter showing a symmetric distribution is silica content $f_{\mathrm{Si}}$.

We now study implications of assigning specific values to parameter subsets. Confining the value of one parameter may narrow the feasible ranges of other parameters and possibly shift the peaks of their marginal distributions. Figure 5b compares consequences of assigning an either low or high value to maximum algal growth rate $\mu_{0}$. The respective choice impacts other parameters to a variable extent. The parameter probably most affected is $K_{\mathrm{light}}$, while impact on parameter *a*, for instance, remains small. Figure 5c illustrates the effects of additionally assuming an either low or high algal silica content $f_{\mathrm{Si}}$. It turns out that this very much affects parameters $\sigma_{0}$ and *a*, while much smaller effects occur for $\lambda_{S}$, for instance. This is a first indication that silica content is in fact a key variable in the overall model concept.

**Figure 6:**
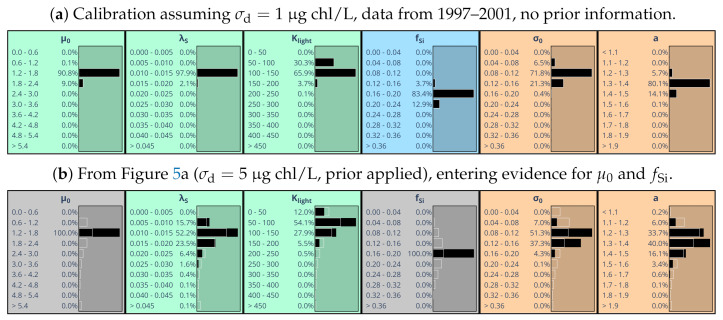
(**a**) Narrow marginal distributions obtained assuming a small observational error $\sigma_{\mathrm{chl}}=1\;\mu$g chl/L, without provision of prior information on feasible parameter values (flat prior). (**b**) Distributions obtained from Figure 5a ($\sigma_{\mathrm{chl}}=5\;\mu$g chl/L, prior applied), evidence for $\mu_{0}$ and $f_{\mathrm{Si}}$ being entered. White contours indicate unconditional distributions.

The widths of marginal parameter distributions depend on the scaling of model-observation discrepancies in the likelihood function Equation (13), achieved by specifying standard deviation $\sigma_{\mathrm{chl}}$. Figure 6a shows the results of choosing $\sigma_{\mathrm{chl}}=1\;\mu$g chl/L instead of 5μg chl/L. The assumed high accuracy of observations prevents divergence of the MCMC process and allows to abandon provision of prior information on parameter distributions. Resulting marginal parameter distributions are very concentrated and located near the maxima of those distributions that were derived assuming larger observational uncertainty in combination with an estimated prior distribution (Figure 5a).

From Equation (38), the effective statistical dimension of feasible parameter space was estimated to be less than three (dof = 2.7). This means that already fixing the values of 2–3 parameters greatly constrains the joint distribution of the six parameters under study. To substantiate this expectation, we constrain Figure 5a by entering again evidence for the two parameters $\mu_{0}$ and $f_{\mathrm{Si}}$, now selecting those values that are most likely according to Figure 6a. As a result (Figure 6b), the marginal distribution for all remaining four variables shrink in reasonable agreement with what one obtains assuming high precision data (Figure 6a).

**Figure 7:**
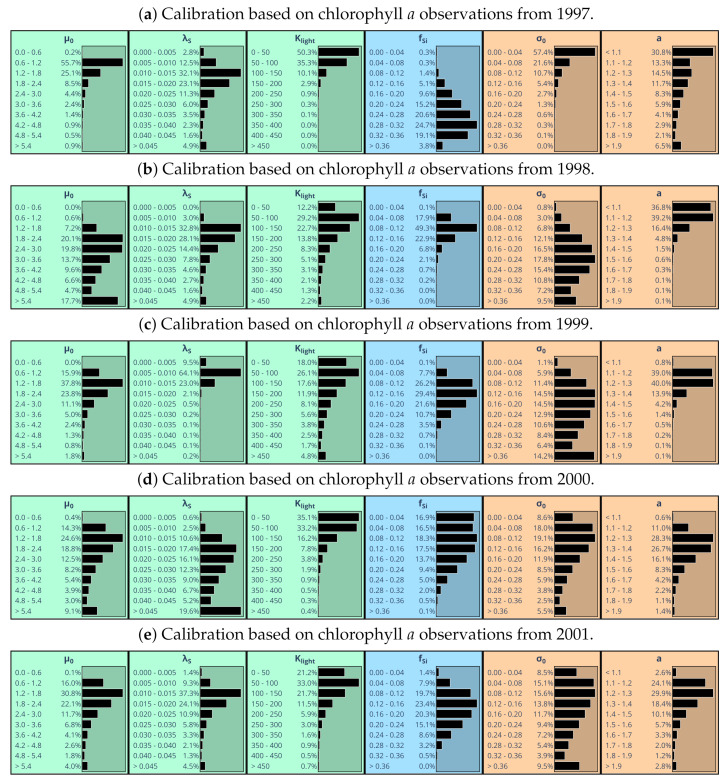
Marginal posterior distributions calibrated using chlorophyll *a* data from different years. The overall setup agrees with that underlying Figure 5a, apart from the different time periods model calibration refers to.

It is interesting to see how posterior parameter distributions differ when calibration is conducted using data from individual years (Figure 7). For 1999 and 2001, posterior marginal distributions of algal silica content $f_{\mathrm{Si}}$ look similar to the one obtained for the full five-year period 1997–2001 (Figure 5a). Relatively low values of algal silica content $f_{\mathrm{Si}}$ specified in agreement with chlorophyll *a* observations in 1998 (Figure 7b) favor the hypothesis that in 1998 algal loss might explain a good deal of chlorophyll *a* concentration variability observed at Geesthacht Weir. The opposite is true for 1997, a year for which the posterior marginal distribution of silica content is shifted toward clearly higher values. For both years 1997 and 1998, marginal posterior distributions hardly depend on whether or not silica observations were used in addition to chlorophyll *a* observations (not shown). This finding holds also for 1999 and 2001. In 1997, silica observations are available only in autumn.

### 5.4. Bayesian Network Assuming Simplified Parameter Interrelationships

So far, we obtained marginal distributions by sampling directly from the MCMC output. However, using advanced BN software with a graphical user interface can very much ease exploration of parameter dependences. Unfortunately, for a larger number of parameters, representation of the joint probability by means of a BN with all edges being kept becomes prohibitive due to the high dimensionality of the conditional probability tables needed. Resolving marginal distributions with a lower number of bins reduces this dimensionality. To avoid coarse resolution, a representation assuming a reduced number of connecting edges often would be the preferred option. However, eliciting the proper simplified structure of a BN [10] is much more difficult than just specifying conditional probability tables for an already given dependence structure. Here, we use Gaussian graphical modeling (Section 4.3.1) as an auxiliary technique, although this concept must be clearly distinguished from the BN approach.

**Figure 8:**
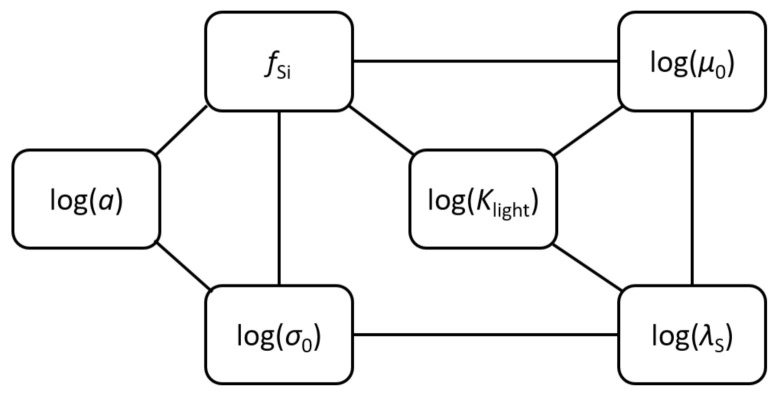
GGM fitted to parameter combinations that proved successful for the years 1997–2001. In the GGM, 6 out of 15 undirected edges representing partial correlation were discarded.

Just as principal component analysis, Gaussian graphical modeling relies on the parameter correlation matrix. An undirected GGM represents correlations (partial correlations) between pairs of variables that are not mediated by any third variables. The idea is to adjust the posterior parameter correlation matrix in a way that generates zero partial correlations. Statistical relevance of any further truncation of the GGM is assessed statistically in the light of existing data. Here, we applied a more qualitative concept, looking for a graph in which all missing edges are clearly less relevant than those maintained (see Section 4.3.1). In the truncated graph we found in agreement with this criterion (Figure 8), 6 out of 15 edges of the saturated graph could be removed.

Table 3 shows the original correlation matrix S of feasible parameter combinations, EOFs of which were displayed in Figure 4. Note the particularly strong correlations (either positive or negative) between algal silica content $f_{\mathrm{Si}}$ and the two algal loss related parameters log(a) and $\log(\sigma_{0})$ (see Equation (8)). Table 3 compares S with correlation matrix V, simplified to agree with the graphical structure shown in Figure 8. The iterative proportional fitting algorithm [48] was applied for adjusting matrix V to conform to this independence structure. Numbers in bold type correspond to edges that were maintained. These correlations generally remain unchanged (see [48]).

Table 3 also shows partial correlation matrices $S_{p}$ and $V_{p}$. Parameters $f_{\mathrm{Si}}$ and log(a) provide an example of how much correlation and partial correlation can differ. Partial correlations that correspond with edges missing in Figure 8 assumed zero values. Other partial correlations just changed their strengths, thereby adapting to the elimination of some mediating variables.

To assess implications of fitting the graphical model, we quantify posterior parameter dependences in terms of the percentages of uncertainty (variability) of every single parameter that can be modeled as a linear function of all other parameters. Table 4 shows these explained variances as derived from S and V, respectively. Generally high values conform to the low dimensionality (dof = 2.7) of the posterior parameter space. Small differences between the results from either S or V are in favor of the simplified GGM. Moreover, the leading EOFs obtained from V resemble those for S in Figure 4 (not shown).

Interpretation of the GGM in Figure 8 is the following. Assume, for instance, that parameters $f_{\mathrm{Si}}$ and $\sigma_{0}$ were given. According to the GGM, this would block all interaction between *a* and the remaining three parameters. Interaction means that changes of any of the three parameters $\mu_{0}$, $\lambda_{S}$ and $K_{\mathrm{light}}$ could compensate for effects of changing *a* and vice versa. Given fixed values for $f_{\mathrm{Si}}$ and $\sigma_{0}$, this mechanism would be suppressed.

**Figure 9:**
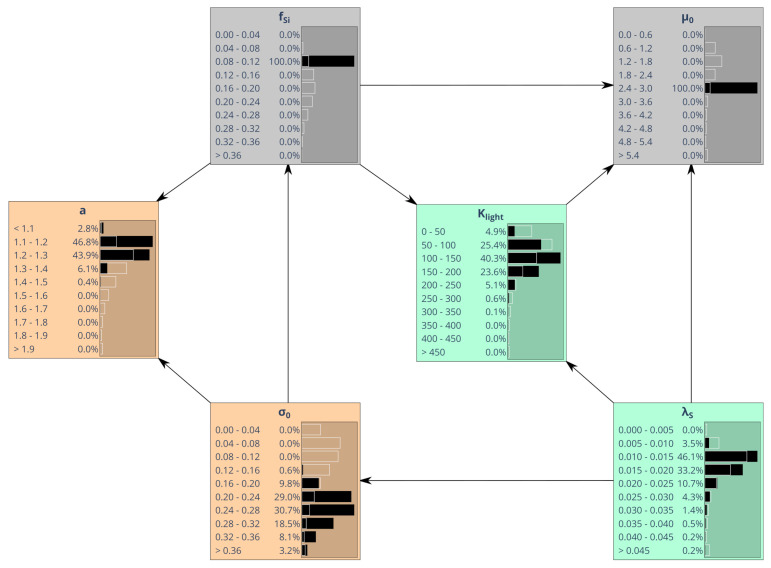
A BN with directed edges only where undirected edges exist in the GGM (Figure 8). The BN is shown in a state after evidence for both $\mu_{0}$ and $f_{\mathrm{Si}}$ was entered. Calculations were performed using Netica. Conditional marginal distributions obtained from the truncated BN well reproduce those shown in Figure 5c. White contours indicate unconditional distributions.

Figure 9 shows a BN with directed edges replacing undirected edges of the GGM in Figure 8. Generally, it is not possible to exactly translate a GGM into a directed BN. Conversely, given any BN, a corresponding conditional independence graph can be derived by first connecting all joint predecessors (parent nodes) of all child nodes. Then, all directed edges are converted into undirected ones, giving the so-called moral graph [76]. The moral graph derived from the BN in Figure 9, for instance, would also contain an edge between $\lambda_{S}$ and $f_{\mathrm{Si}}$, as these two nodes have joint children $K_{\mathrm{light}}$ and $\mu_{0}$. Such edge is missing in the GGM in Figure 8. Hence, the GGM tends to be more restrictive than the BN and it can therefore be expected that the simplified BN (6 edges out of 15 edges were removed; maximum number of parents is 3 instead of 5 for the saturated graph) behaves similar to the saturated BN.

To substantiate this agreement, Figure 9 replicates the experiment shown in the upper panels of Figure 5c, assigning small values to both maximum growth rate $\mu_{0}$ and silica content $f_{\mathrm{Si}}$. Marginal distributions of the remaining four parameters show reasonable (although not perfect) agreement with Figure 5c (calculations were performed using Netica; https://www.norsys.com, accessed on 29 September 2021).

### 5.5. Accelerated Sampling via Delayed Acceptance

As mentioned above, the large required number of model runs to obtain the presented results can be reduced by delayed acceptance MCMC sampling. Instead of directly using the surrogate for the cost *ℓ* with fixed ζ, we take a step in-between and construct a function-valued surrogate model. Multiple surrogates ${\tilde{z}}_{k}\left(x\right)$ are built, where each maps the input x to one weight $z_{k}\left(x\right)$ in the KL expansion. A surrogate ${\tilde{f}}_{i}\left(x\right)\equiv \tilde{f}\left(t_{i};x\right)$ for the model output is then given by replacing $z_{k}\left(x\right)$ by ${\tilde{z}}_{k}\left(x\right)$ in (22). The according surrogate $\tilde{\ell }\left(y;x,\zeta \right)$ for the cost function uses ${\tilde{f}}_{i}\left(x\right)$ instead of $f_{i}\left(x\right)$ in (35). Dependencies on ζ are kept exact in this approach. The main algorithm proceeds in the following steps:

1. Construct a GP surrogate for the $L_{2}$ cost function on a space-filling sample sequence over the whole prior range.
2. Refine the sampling points near the posterior’s mode by Bayesian global optimization with the $L_{2}$ cost surrogate.
3. Train a multi-output GP surrogate for the functional output z(x) on the refined sampling points.
4. Use the function-valued surrogate for delayed acceptance in the MCMC run.

For all GP surrogates, we use a Matern 5/2 kernel for $k(x,x^{\prime })$ together with a linear mean model for m(x). For step 4, we use Gibbs sampling and the surrogate for z(x) yielding the full output y(t,x) rather than only the $L_{2}$ distance to a certain reference dataset. The idea to refine the surrogate iteratively during MCMC had to be abandoned early. The problem is that detailed balance is violated as soon as the surrogate proposal probabilities change when modifying the GP regressor with a new point. In the following application cases, we compare a usual MCMC evaluation using the full model to MCMC with delayed acceptance using the GP surrogate together with the KL expansion/functional PCA (GP+KL) in the output function space.

First, we test the quality of the algorithm on a toy model given by

(39) $$y\left(t,x\right)=x_{1}\sin\left({\left(t-x_{2}\right)}^{3}\right).$$

**Figure 10:**
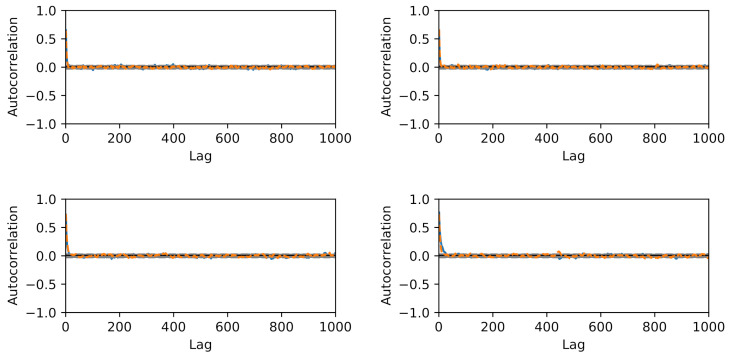
Autocorrelation over lag in MCMC steps for inputs $x_{1}$ (solid) and $x_{2}$ (dashed) in the toy model. (**Top**): Gaussian likelihood, (**bottom**): hierarchical model. (**Left**): full MCMC, (**right**): delayed acceptance MCMC with GP+KL surrogate.

**Figure 11:**
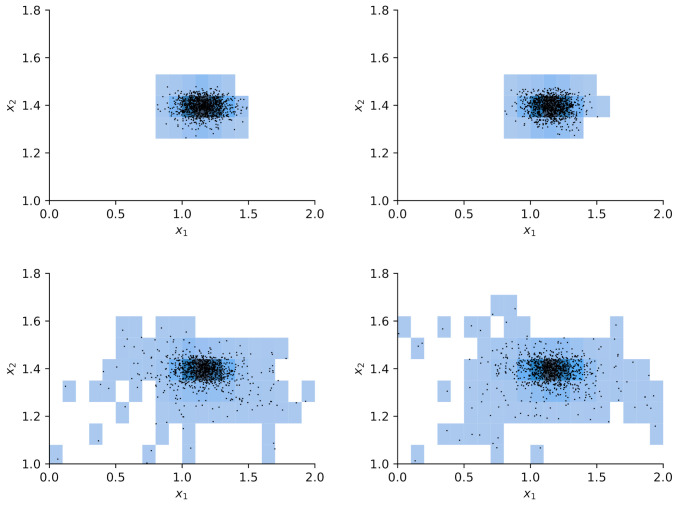
Posterior distribution of calibrated parameters x in (39). (**Top**): Gaussian likelihood, (**bottom**): hierarchical model. (**Left**): full MCMC, (**right**): delayed acceptance MCMC with GP+KL surrogate.

We choose reference values $x_{1}=1.15,\;x_{2}=1.4$ to test calibration of x against the according output $y^{\mathrm{ref}}\left(t\right)\equiv y\left(t,x^{\mathrm{ref}}\right)$ and add Gaussian noise of amplitude σ=0.05. A flat prior is used for x. For the hierarchical model case (34), we choose a starting guess of ζ=2 for the norm’s order and a Gaussian prior with $\sigma_{\zeta }=0.5$ around this value together with a positivity constraint. The initial sampling domain is the square $x_{1},x_{2}\ldots \left(0,2\right)$. The comparison between MCMC and delayed acceptance MCMC is made once for fixed ζ=2 (Gaussian likelihood) and then for a hierarchical model with a random walk also in ζ. The respective Markov chain with 10.000 steps has a correlation length of ≈10 steps (Figure 10) and yields a posterior parameter distribution for $\left(x_{1},x_{2}\right)$ depicted in Figure 11.

**Figure 12:**
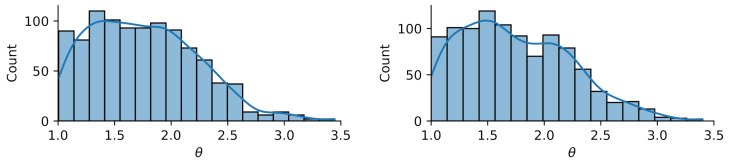
Posterior distribution of the fractional order ζ in the loss function with $\ell^{\zeta }$ norm. (**Left**): full MCMC, (**right**): delayed acceptance MCMC with GP+KL surrogate.

The results in Figure 11 show good agreement in the posterior distributions of full MCMC and delayed acceptance MCMC. Compared to the case with fixed ζ=2, the additional freedom in ζ in the hierarchical model leads to further exploration of the parameter space. The posterior of ζ according to the Markov chain is given in Figure 12. The similarity to the prior distribution shows that the data does not yield new information on how to choose ζ.

**Figure 13:**
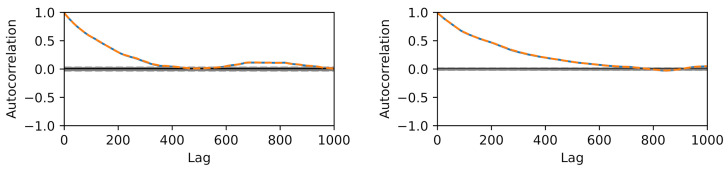
Autocorrelation over lag in MCMC steps for inputs $K_{\mathrm{light}}$ (solid) and $\mu_{0}$ (dashed) in the riverine diatom model. (**Left**): full MCMC, (**right**): delayed acceptance MCMC with GP+KL surrogate.

**Figure 14:**
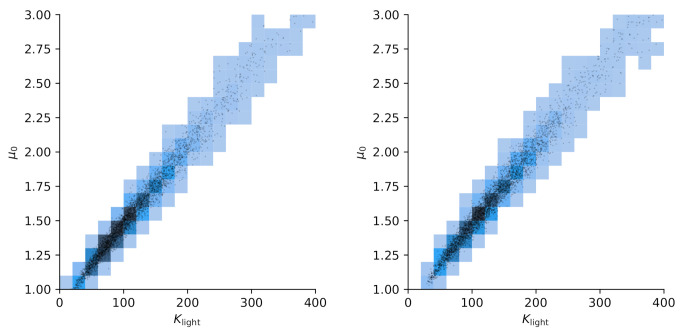
Posterior distribution of calibrated parameters for the riverine diatom model. (**Left**): full MCMC, (**right**): delayed acceptance MCMC with GP+KL surrogate.

The construction of a reliable Gaussian process surrogate for the full six-dimensional input space of the diatom model has not been successful due to the excessive number of support points. This is why we limit the present analysis to only two input parameters, namely $x_{1}=\theta_{3}=K_{\mathrm{light}}$ and $x_{2}=\theta_{1}=\mu_{0}$. As in the case of the toy model, we use 10.000 steps in the Markov chain. Results for autocorrelation and posterior samples using the full model versus delayed acceptance are shown in Figure 13 and Figure 14. The correlation time of ≈500 steps is much larger than in the toy model, and the decay of the autocorrelation over the lag roughly matches between the two approaches. Delayed acceptance sampling produces similar posterior samples in Figure 14 at about one third of the overall computation time. There one also sees the issue of high correlation between $K_{\mathrm{light}}$ and $\mu_{0}$ in the posterior of the calibration, making Gibbs sampling inefficient in this particular case.

## 6. Discussion

### 6.1. The Case Study Example

Our case study considered a simple model with only few parameters that nevertheless reproduced chlorophyll *a* observations at Geesthacht Weir reasonably well. However, even a good fit does not prove a model’s truth [1,3,5]. The fact that even complex environmental models drastically simplify the natural system has attracted much scepticism, e.g., [77]. Model design always relies on certain presumptions. Hornberger and Spear [78] considered their simple model for the Peel Inlet a speculative scenario that presumes phosphorus controls algae growth. An alternative scenario putting nitrogen at the heart of the analysis was reported by Humphries et al. [79]. Similarly, the model in the present case study was based on the hypothesis that lack of silica might explain sudden drops of chlorophyll *a* concentration observed at station Weir Geesthacht. Model calibration shed some light on how (within the a priori specified model structure!) silica limitation and a temperature dependent loss rate could provide competing concepts to explain variability of chlorophyll *a* observations.

An indication in favor of the basic model concept is the fact that when small observational errors were assumed, a disregard of prior knowledge about model parameter values did not let MCMC simulations produce infeasible parameter combinations (Figure 6a). According to van Straten [80] ‘...one may question, whether a model is actually well-structured if the use of parameter constraints is the only way to avoid nonfeasible solutions’. Another vague indication in favor of the underlying model concept is the fact that posterior parameter distributions did not much depend on whether or not silica observations were used in the process of model calibration (not shown). On the other hand, the model consistently failed to reproduce a sharp late spring chlorophyll *a* increase, so that in each year silica consumption had to be abandoned for a 1–2 week period. This model deficiency could not be fixed by any choice of parameters (not discussed in this paper), which confirms that the model’s structure is specific and cannot be adjusted to any arbitrary time series.

One must be aware that simple (or even complex) models neglect many external factors that potentially impact time series observed in nature. Observations from different years cannot necessarily be treated like the outcomes of repeated well-defined experiments. External factors not considered in the model (there are plenty of them) might differ in different years. Discussing an exceptionally high chlorophyll *a* concentration in the River Rhine in 2011, Hardenbicker et al. [37] hypothesize a high growth potential of phytoplankton which, however, most of the time is suppressed by some other environmental factor. From a modeler’s perspective, Waylett et al. [38] found that particularly high spring chlorophyll *a* concentrations in one year could not be explained by physico-chemical factors in their model. They suggested variable strengths of grazing loss rates, possibly depending on over-winter survival rates of benthic filter feeders, being the most feasible explanation for such interannual differences. According to Figure 7, our analyses for the two years 1997 and 1998 suggest an either larger (1997) or smaller (1998) impact of silica limitation, assuming an either high (1997) or low (1998) algal silica content. One must be careful to prematurely attribute such differences for individual years to just the model being overfitted.

A proper choice between the two explanations of chlorophyll *a* variability (either silica limitation or increased algae loss rate) might gain importance as soon as the model would be run in a predictive mode. However, extrapolation of a simplified model into a domain of unobserved environmental conditions would be a questionable enterprise. The goal of the present study in support of past data interpretation was rather to improve the description of imponderabilities that remain after model calibration.

We assessed model performance in terms of a squared-error loss function (Equation (13)). A quadratic measure, penalizing in particular large discrepancies, is suitable to highlight problems with the simulation of major short-term changes that characterized the chlorophyll *a* time series under study, for instance. Using the squared-error loss function might have been less revealing if the general model performance had been worse (that is why we modified the model during the short periods in May/June). In case of generally strong model data discrepancies, a linear measure of model data misfit could have been more adequate.

### 6.2. MCMC in Relation to GLUE and BMC

Modeling eutrophication in the Peel Inlet, Hornberger and Spear [78] and Hornberger [81] formulated a set of six behavioral conditions to discriminate between simulations being either successful or unsuccessful in mimicking key aspects of the system’s evolution. Spear and Hornberger [82] found separation induced correlations between model parameters to not exceed 0.23, which is why the authors did not embark on a deeper analysis of the correlation matrix. According to Spear [4], conventional multivariate analyses proved to be also unhelpful in other studies using the same approach. In our study, correlations were found to be much higher (see Table 3). We presume that this relates to (a) our model being much more controlled by observations and (b) the huge number of successful simulations ($10^{6}$ in our study) affordable with today’s computer power.

The aforementioned studies motivated further developments leading to the GLUE (generalized likelihood uncertainty estimation; [83]) technique, which sometimes is referred to as a pseudo-Bayesian approach. In contrast to MCMC (a formal Bayesian approach), the GLUE approach separates parameter sampling (either uniform Monte Carlo or Latin Hypercube Sampling, for instance) from likelihood evaluation [22]. For higher dimensions, the random sampling makes GLUE computationally more expensive than MCMC using sequential sampling [25]. Random sampling is also used within the Bayesian Monte Carlo (BMC) approach, a method related to GLUE but using a statistically rigorous likelihood function [23]. According to Beven and Freer [22], the advantage of MCMC might diminish when model output likelihood has a complex shape. That seemed not to be the case in our application.

Tan et al. [84] contrasted results of the GLUE and MCMC approaches, assessing uncertainties of nine parameters of a crop model. The authors did not address, however, an explicit specification of parameter interactions according to the posterior joint distribution. The same holds for a comparative assessment of the two approaches conducted by Li et al. [85], referring to two conceptual hydrological models, or Camacho et al. [25], reporting a study on the calibration of a hydraulic or hydrodynamic model using synthetic data. Our study focused on parameter interactions, and we believe that for that purpose the many samples obtainable from MCMC are a key advantage when it comes to filling a higher dimensional parameter space. Using BMC to calibrate nine parameters of a simple water quality model, Dilks et al. [23] found that approximately 60% of model output uncertainty could be related to covariances between model input parameters. For a lake modeling example, Fedra et al. [1] found that focusing on meaningful simulations did not much constrain individual parameters. However, in higher dimensions, they found clustering of successful parameter combinations.

Referring to a binary classification of model runs in terms of simulations being successful (‘passes’ or ‘behaviours’) or not, Spear et al. [86] described the interactions between parameters that gave rise to passes by a tree-structured estimation technique. Studying an example from groundwater pathways modeling, the authors found discontinuous localized regions, interactions of which were not reflected in a linear correlation matrix. A similar feature could not be recognized in our study. The reason might be that our analysis was based on a continuous goodness-of-fit index rather than a sharp binary classification.

### 6.3. Benefit from Using BNs

Discussing half-saturation constants, Mulder and Hendriks [87] warn that simultaneous calibration of a whole set of model parameters might not reveal the true values one would obtain in the laboratory. Similarly, Brun et al. [5] emphasize that fixing selected parameters will usually bias the estimates of other parameters. However, experimental data are sparse and often not representative for the overall description of a complex natural system. An at least approximate description of the joint posterior distribution of all parameters offers a way out of this dilemma. A BN representing this distribution enables users to explore the extent to which selecting values for any subset of parameters constrains the marginal distributions of all other parameters (see Figure 5), thereby explicitly addressing the concerns raised by Brun et al. [5].

Marginal distributions constrained by available evidence on some of the model parameters can also be calculated directly from MCMC output, without an involvement of specialized BN software. The advantage is that high-dimensional conditional probability tables that hamper the analysis need not be specified. However, using BN software with a graphical user interface provides a more convenient approach. BN software is also needed when aiming at a simplified representation of posterior parameter interactions. Structural analysis might suggest some kind of stepwise calibration of different process related modules of a model. We demonstrated how in this context graphical Gaussian modeling could be helpful, given that parameter uncertainty distributions are reasonably well represented by a simple linear correlation matrix. Although undirected and directed graphs cannot be directly translated into each other, the undirected graph seems nevertheless more informative than conventional principal component analysis, for instance.

Parameter correlations documented in the BN represent alternative model structures, an example of the equifinality which, according to Beven and Freer [22], may occur already for moderate model complexity. Many authors discussed that a model accommodating such overparameterization, not pretending existence of a single true model, may even be useful, e.g., [1,3,22,88]. Given the model structure we used, the aspect worst controlled by data is decreasing chlorophyll *a* concentrations explained by either algae growth limited by lack of silica or a strong algal loss rate. According to Figure 5c, choosing a relatively low algal silica content $f_{\mathrm{Si}}$ (and therefore a low depletion of the silica reservoir) implies a large loss rate $\sigma_{0}$ and a small coefficient *a* governing effects of temperature on the loss rate (e.g., via grazing). By contrast, for a large silica content, the maximum loss rate should be set to a small value; its variation for high temperatures becomes less constrained by the data.

## 7. Conclusions

For a simple model of riverine diatoms, we provided a detailed description of the posterior joint distribution of adequate model parameters, inferred via observations of chlorophyll *a* concentration. We argue that this is the most profound information on a model’s calibration potential users can achieve. Results showed how, within the pre-specified model structure, two different processes affected model output in a very similar way, thereby offering different interpretations of features in the observations. Of course, it must be kept in mind that (like with any other ecosystem model) already choosing the specific model structure vastly simplified the representation of nature by the neglect of many (in fact, the majority of) detailed processes.

Two challenges must be met for the approach we discussed. First, estimation of the joint distribution in a higher dimensional parameter space needs a large number of simulations. MCMC seems more effective than random sampling, avoiding exploration of those regions in parameter spaces that produce unrealistic simulations. As MCMC would still be prohibitive for large models, provision of computationally less demanding surrogate models could offer a way out.

As a possible way to reduce the number of simulations in MCMC, we have illustrated the application of function-valued surrogates to delayed acceptance MCMC for parameter calibration in simple as well as hierarchical Bayesian models. Using a surrogate for the functional output rather than cost function or likelihood is useful for several reasons. Conceptually, it allows introducing additional distribution parameters in Bayesian hierarchical models. Our results demonstrate that it is possible and efficient to perform MCMC with delayed acceptance on such models while keeping dependencies in these additional parameters exact. In particular, the fractional order of the norm appearing in the cost function has been left free, which is useful for robust model calibration. The method was applied to a toy model and the present application with a limitation to two variable input parameters. In both cases, using delayed acceptance with a surrogate for the functional output produced results comparable to using the full model at only about one third of actual model evaluations. Compared to direct surrogate modeling of the cost function, we could also observe an increase in the quality of the predicted cost. This is likely connected to the higher flexibility of modeling weights to multiple principal components with Gaussian processes with individual hyperparameters.

The described approach is not immune to the curse of dimensionality. On the one hand, the number of required GP regressors grows linearly with the effective dimension of the output function space. Since evaluation is fast and parallelizable, this is a minor issue in practice. On the other hand, increasing the dimension of the input space soon prohibits the construction of a reliable surrogate due to the required number of training points to fill the parameter space. In such cases, the preprocessing overhead is expected to outweigh the speedup of delayed acceptance MCMC for either functional or scalar surrogates. More detailed investigations will be required to give quantitative estimates on this tradeoff. In the future, it may further be of interest to leverage the uncertainty information given by the GP regressor to decide in which regions of parameter space the surrogate is reliable enough to provide a delayed acceptance proxy.

The second challenge is a convenient representation of the joint posterior parameter distribution. BN software estimates a set of conditional probability tables that represent results from the model calibration exercise. While direct sub-sampling the original data avoids technical problems with high-dimensional conditional probability tables, BN technology enables a simplified representation of parameter interactions. For large models, a graph of model parameter interactions could possibly be assembled from a number of sub-graphs dealing with parameters of different modules of a process-oriented model. Proving feasibility of such an approach is left to further research.

## Figures and Tables

**Table 1:** Parameter values assumed to be exceeded with probability 10 percent (1-*P*^☆^, see Equation (15)).

	$\mu_{0}$	$\lambda_{S}$	$K_{\mathrm{light}}$	$f_{\mathrm{Si}}$	$\sigma_{0}$	*a*
	Equation (4)	Equation (5)	Equation (6)	Equation (2)	Equation (8)	
$\theta_{k}^{★}$:	3.5	0.05	500	0.4	2.0	2.0
	d${}^{-1}$	${\left(m\;\mu g\;\mathrm{Chl}\right)}^{-1}$	W/m${}^{2}$	mg Si/mg C	d${}^{-1}$	-

**Table 2:** Parameters underlying Figure 3 (cost: first term in Equation (13), evaluated for 1997–2001; prior: second term in Equation (13)).

	$\lambda_{S}$	$K_{\mathrm{light}}$	$f_{\mathrm{Si}}$	*a*	$\mu_{0}$	$\sigma_{0}$	Color in Figure 3	Cost/Prior
	${\left(m\;\mu g\;\mathrm{Chl}\right)}^{-1}$	W/m${}^{2}$	mg Si/mg C	-	d${}^{-1}$	d${}^{-1}$		
Minimum cost function	0.0118	41.9	0.168	1.25	1.19	0.150	black	14.2/4.8
Max. chlorophyll *a* on 11 May	0.0054	216	0.145	1.34	1.89	0.156	green	23.6/6.6
Max. chlorophyll *a* on 10 July	0.0172	2.8	0.214	1.50	0.62	0.026	brown	26.2/4.8
Min. chlorophyll *a* on 31 July	0.0081	29.8	0.296	2.82	0.71	0.011	red	25.7/5.9

**Table 3:** Up: Correlation matrix S of feasible model parameters (upper triangle) and correlation matrix V fitted to comply with the GGM shown in Figure 8 (lower triangle). Down: Corresponding matrices $S_{p}$ and $V_{p}$ of partial correlations. Numbers in **bold type** correspond to edges that were maintained in the GGM.

V∖S						
	$\log(\lambda_{S})$	$\log(K_{\mathrm{light}})$	$f_{\mathrm{Si}}$	log(a)	$\log(\mu_{0})$	$\log(\sigma_{0})$
$\log(\lambda_{S})$		**0.11**	−0.21	−0.10	**0.69**	**0.10**
$\log(K_{\mathrm{light}})$	**0.11**		**−0.07**	0.08	**0.62**	−0.02
$f_{\mathrm{Si}}$	−0.23	**−0.07**		**0.74**	**−0.50**	**−0.94**
log(a)	0.01	−0.04	**0.74**		−0.29	**−0.88**
$\log(\mu_{0})$	**0.69**	**0.62**	**−0.50**	−0.26		0.37
$\log(\sigma_{0})$	**0.10**	0.06	**−0.94**	**−0.88**	0.40	
$V_{p}\setminus S_{p}$						
	$\log(\lambda_{S})$	$\log(K_{\mathrm{light}})$	$f_{\mathrm{Si}}$	log(a)	$\log(\mu_{0})$	$\log(\sigma_{0})$
$\log(\lambda_{S})$		**−0.69**	0.05	−0.03	**0.83**	**−0.12**
$\log(K_{\mathrm{light}})$	**−0.65**		**0.14**	0.11	**0.84**	−0.01
$f_{\mathrm{Si}}$	0	**0.20**		**−0.54**	**−0.30**	**−0.89**
log(a)	0	0	**−0.46**		−0.12	**−0.79**
$\log(\mu_{0})$	**0.82**	**0.83**	**−0.25**	0		−0.08
$\log(\sigma_{0})$	**−0.13**	0	**−0.87**	**−0.77**	0	

**Table 4:** Portions of parameter variability that can be modeled as a linear function of all other five parameters. Values are specified for correlation matrices S and V (cf. Section 5.4).

	log($\lambda_{S})$	log($K_{\mathrm{light}})$	$f_{\mathrm{Si}}$	log(*a*)	log($\mu_{0}$)	log($\sigma_{0}$)
S:	74%	72%	94%	84%	89%	96%
V:	73%	69%	93%	83%	87%	96%

## Data Availability

Data are available on Zenodo https://doi.org/10.5281/zenodo.5773864 (accessed on 29 September 2021) and on request to the authors.
